# Evidences on the Ability of Mycorrhizal Genus *Piloderma* to Use Organic Nitrogen and Deliver It to Scots Pine

**DOI:** 10.1371/journal.pone.0131561

**Published:** 2015-07-01

**Authors:** Jussi Heinonsalo, Hui Sun, Minna Santalahti, Kirsi Bäcklund, Pertti Hari, Jukka Pumpanen

**Affiliations:** 1 Department of Food and Environmental Sciences, University of Helsinki, Helsinki, Finland; 2 Department of Forest Sciences, University of Helsinki, Helsinki, Finland; Friedrich Schiller University, GERMANY

## Abstract

Ectomycorrhizal (ECM) symbiosis has been proposed to link plant photosynthesis and soil organic matter (SOM) decomposition through the production of fungal enzymes which promote SOM degradation and nitrogen (N) uptake. However, laboratory and field evidence for the existence of these processes are rare. *Piloderma* sp., a common ECM genus in boreal forest soil, was chosen as model mycorrhiza for this study. The abundance of *Piloderma* sp. was studied in root tips and soil over one growing season and in winter. Protease production was measured from ectomycorrhiza and soil solution in the field and pure fungal cultures. We also tested the effect of *Piloderma olivaceum* on host plant organic N nutrition in the laboratory. The results showed that *Piloderma* sp. was highly abundant in the field and produced extracellular proteases, which correlated positively with the gross primary production, temperature and soil respiration. In the laboratory, *Piloderma olivaceum* could improve the ability of *Pinus sylvestris* L. to utilize N from extragenous proteins. We suggest that ECM fungi, although potentially retaining N in their hyphae, are important in forest C and N cycling due to their ability to access proteinaeous N. As *Piloderma* sp. abundance appeared to be seasonally highly variable, recycling of fungal-bound N after hyphal death may therefore be of primary importance for the N cycling in boreal ecosystems.

## Introduction

The balance between soil organic matter (SOM) decomposition and litter accumulation is of primary importance to the boreal forest carbon (C) budget. Boreal forest soil is one of the largest terrestrial C pools [1] and any changes in the C balance in this ecosystem could have global consequences [2]. Boreal forests are typically dominated by trees that form symbiosis with ectomycorrhizal fungi (ECM) and by understory vegetation that forms ericoid mycorrhizal (ERM) associations [3]. In the ECM symbiosis, the plant delivers C compounds fixed during photosynthesis to the ECM which in turn assists the host plant in nutrient and water uptake [3]. ECM also play a role in protecting the plant against attack by harmful microbial pathogens [3]. In ECM ecosystems, SOM accumulates and nutrients are present in an organic form resulting in an ‘organic nutrient economy’ [4]. In order to acquire N and P, trees translocate large amounts of carbohydrates belowground to microbes and particularly ECM, which links soil processes to forest productivity [3]. Surprisingly ECM and ERM ecosystems have been shown to store almost twice as much C per unit of soil N than arbuscular mycorrhizal ecosystems, indicating that the type of mycorrhizal symbiosis is an important determinant for soil C storage [5]. The incorporation of interactions between the C and N cycles into climate system models has therefore been recommended [6].

Recently it has been shown that the decomposition of recalcitrant, slow-cycling SOM pool is accelerated by easily available sources of energy, such as cellulose or glucose [7,8], in a process called priming [9]. The easily utilizable carbohydrates facilitate the production of extracellular enzymes needed for SOM decomposition by microorganisms. The priming effect is not only dependent on a C source but also on other factors, including the nutritional status of the soil, and in some cases, neither priming nor even ‘negative priming’ has been observed [9]. Recent studies on boreal forest soils have shown that the priming effect is connected to N uptake, and as boreal forests are often N-limited, N scavenging from complex organic molecules has been proposed to be the main trigger of the priming effect [10, 11]. There is increasing body of evidence, which suggests that ECM fungi play a key role in priming, soil organic N transformations and SOM decomposition [12–14].

In contrast to wood-decomposing fungi, ECM fungi are not known to produce significant quantities of peroxidases (e.g. manganese peroxidase, MnP) that degrade complex, lignin-containing structures. However, there is evidence that MnP-like peroxidases may be produced by the common ECM genus *Cortinarius*, and class II peroxidase-encoding genes have been identified in a wide range of ECM fungi [15]. High peroxidase activity and DNA quantity from *Cortinarius* species were co-localized in boreal forest soil [13]. Recently, Rineau *et al*. [16] demonstrated that ECM fungus *Paxillus involutus* may degrade SOM using a Fenton-reaction based mechanism in a similar way as brown-rot fungi. Laccase, a non-specific oxidative enzyme, is known to be produced by many fungi, including ECM [17–19]. Multiple laccase genes are encoded in the genome of *Piloderma* sp. [20] and their expression was related to N availability [17]. Heinonsalo *et al*. [19] screened 23 fungal strains and showed that laccase activities in *Piloderma olivaceum* pure culture were triggered by the addition of humus and Scots pine (*Pinus sylvestris*) sawdust. The measured laccase activities of *P*. *olivaceum* were the highest of the five studied mycorrhizal strains and were in the same range as the laccase activities of litter-decomposing and white-rot fungi. Laccase activity is also commonly observed in ECM root tips in the field [21].

Proteolytic enzyme activity is necessary if recalcitrant soil organic N pool is to be utilized, as there are large quantities of protein-like compounds in SOM [22]. Stevenson [23] reported that 30–35% of soil N consists of extracellular proteins, bound in an organic matrix. Already in the 1980’s, ECM fungi were divided into ‘protein-fungi’ and ‘non-protein fungi’ according to their ability to utilize protein and amino acids as sole N source [3, 24]. Chalot & Brun [25] provided evidence that ECM fungi have the ability to use proteins as N source and demonstrated that amino acids are an important N source for ECM fungi. More field evidence was anticipated to be required in order to confirm these results [25]. The main focus of the studies of Abuzinadah & Read [24] and Chalot & Brun [25] was to investigate the N sources used by ECM fungi. The effect of N source on the priming effect and SOM decomposition were not considered at that time. However, the link between SOM degrading enzyme activity and proteolytic activity is now considered an important issue, and needs to be taken into account when the effect of increasing forest productivity (incl. higher photosynthetic rates under high CO_2_ environments) on soil C balance are considered. Although recent studies propose significant contribution of ECM fungi on SOM transformation processes [11, 13–15, 26, 27], experimental evidence on the existence of these processes on a field scale are scarce, and more knowledge is required in order to establish a link between the forest NPP, SOM decomposition and ECM fungi.

The aim of this study was to investigate the ability of *Piloderma* sp., a model ECM genus, to produce proteases and to determine its importance in Scots pine organic N uptake. The abundance of *Piloderma* sp. in the field was investigated in relation to protease production, forest productivity and environmental factors. Experimentation ranged from axenic laboratory experiments with *P*. *olivaceum* to seasonal field studies, allowing us to connect laboratory and field findings. We hypothesized that *Piloderma olivaceum* can use organic N sources in order to deliver N to its host plant. We also hypothesized that the abundance and protease production of *Piloderma* sp. depend on host plant productivity and growing season.

## Materials and Methods

### Laboratory experiments

#### The effect of N source on fungal growth, biomass production and nitrogen uptake of host plant in symbiosis with *P*. *olivaceum*

To test the effect of N source on the growth of *P*. *olivaceum* (100% ITS sequence similarity also to *P*. *croceum*) (Fungal Biotechnology Culture Collection, University of Helsinki, Finland; FBCC 1391, see S1 Table) in pure culture, the strain was grown (N = 3) in axenic conditions on three different HA-based ([28]; with no agar) liquid growth media: 1) NH_4_Cl was used as a mineral N source; 2) 90% of elemental N of the NH_4_Cl was replaced by an equal mixture of four common amino acids [29] (aspartic and glutamic acid, glycine and DL-alanine); and 3) 90% of elemental N of the NH_4_Cl was replaced by a protein with no function in the natural environment (Bovine serum albumin, BSA, Sigma Aldrich product no A9418, St. Louis, MO, USA). All three media contained equal amounts of elemental N and volume of the media was 100mL. Cultures were grown without shaking at +28°C. Fungal biomass (g DW) was analyzed after 90-day incubation to determine the role of N source on the growth of *P*. *olivaceum*.

To investigate the fungal effect on host plant N uptake, Scots pine (*P*. *sylvestris*, L.) seeds were surface sterilized using 30% H_2_O_2_, washed in sterile distilled water and germinated on 1.6% glucose agar to detect any microbial contaminants. After germination, the seedlings were transferred to sterile glass tubes (diameter 25 mm, height 200 mm) containing 10 mL BW-agar [30] and oven-burned Leca clay pellets (see details in [31]). After a four-month growth period, half of the seedlings were inoculated with *P*. *olivaceum* agar plugs (FBCC 1391, see S1 Table), the other half were inoculated with sterile agar plugs. Once the inoculated seedlings were mycorrhizal, all the seedlings were divided into three N fertilization treatments (N = 5): 1) a treatment without extra N, only water added; 2) a treatment supplemented with 12 mg N in the form of an amino acid mix (aspartic and glutamic acid, glycine and DL-alanine in equal proportions); and 3) a treatment supplemented with 12 mg N in the form of BSA protein. The original BW media contain NH_4_^+^ and NO_3_^-^ that allowed adequate growth for seedlings during the first months. The seedlings were watered regularly to maintain growth. The seedlings were harvested after a further 3.5-month growth and the biomass and N concentration of the needles were determined. The total growth period was nine months.

#### Protease production of fungal strains

Fungal cultures (S1 Table) obtained from the FBCC culture collection, and isolates from Scots pine root tips from Hyytiälä Forest Station in South-Eastern Finland (61°51'N, 24°17'E, see Soil sampling and morphotype analysis) were tested for the production of proteases. The cultures were grown on Hagem’s agar (HA) media [28] for 7 weeks and a 4 mm diameter round piece of culture (containing agar) was used for analyses (N = 3 from different plates). Protease quantity was analyzed using the PF0100 Protease Fluorescent Detection Kit (Sigma Aldrich, St. Louis, MO, USA) according to the manufacturer’s recommendations, in which an agar plug with a living mycelium was used instead of a liquid sample. An agar plug without fungal mycelium was used as a negative control. The measured fluorescent counts were compared to a 0 to 200 ng trypsin standard curve and results were given as ng protease per cm^2^ (surface area of the culture).

### Field experiment

#### Soil sampling and morphotype analysis

Monthly soil core sampling was performed from a 60-year-old Scots pine forest in Hyytiälä SMEAR II Forest Station (61°51'N, 24°17'E) [32] between March and October 2011. The field sampling was done with permission of the University of Helsinki, given by prof. Jaana Bäck. Three separate plots with distance ranging from 50 to 60 meters from each other were selected within the forest stand. Within each plot, five soil cores with a diameter of 5 cm and one meter distance from each other were taken and stored at +4°C, and analyzed within three days. The soil cores from March were kept frozen (-20°C) until analyzed as the soil was frozen at the time of the sampling. No data is available from April, when the snow cover melted.

Scots pine root tips present in the humus layer were separated from the soil cores and counted at each sampling time. The average depth of the humus layer was 2.95 cm. The quantity of the bright yellow *Piloderma* sp. morphotype was determined under a stereo microscope and compared to the number of all other non-identified ECM morphotypes. The humus layer of five cores sampled at each plot were pooled resulting in three replicates (N = 3) at one time point and used later for DNA extraction and pyrosequencing.

#### Measurement of protease production in mycorrhiza and soil solution

From each soil core sample (see above), ten randomly selected ECM root tips were taken for protease production measurements. The measurement was done according to manufacturer’s recommendations (see previous chapter) except that individual ECM root tips were placed into reaction tube, instead of liquid sample. The proportion of yellow *Piloderma* sp. morphotype was recorded. From the soil core, homogenized humus was placed into 500 μL filter tubes and soil solution was collected after 30 min centrifugation (15 700 × g) [19]. Protease production measurement was performed according to manufacturer’s recommendations (see previous chapter). Data from root tip and soil samples were calculated to represent the whole soil core, and extrapolated to surface area of soil (m^2^) to be able to compare ECM root tip and soil protease production.

#### DNA extraction, amplification of ITS2 region and pyrosequencing

Soil samples were thawed at +4°C and homogenized by hand after removing rocks, large particles and roots. Genomic DNA was extracted from 0.25 g of each homogenized soil sample using the ‘PowerSoil DNA Isolation Kit’ (MoBio Laboratories, Carlsbad, CA, USA) according to the manufacturer’s instructions. The bead beating homogenization was done with FastPrep instrument (MP Biomedicals, LLC, France) with 4 ms^-1^ for 30 seconds. Extracted DNA was quantified with a NanoDrop 1000 spectrometer (NanoDrop Technologies, Wilmington, DE, USA) and adjusted to a final concentration of 10 ng μl^-1^.

PCR reactions were performed in triplicate for each sample to minimize PCR biases. The fungal-specific primer pairs gITS7 (containing the 454 pyrosequencing A-adapter) and ITS4 (containing the 454 pyrosequencing B-adapter and a 6 base pair barcode) were used to amplify ITS2 region [33] (S2 Table). A total of 30 ng of template DNA was used in a 25-μl PCR reaction, which included Phusion High-Fidelity DNA polymerase (Thermo Scientific, Vantaa, Finland). The following cycling parameters were used: 1 cycle of 98°C for 30 s, 23 cycles of 98°C for 10s, 56°C for 30 s and 72°C for 20 s followed by a 5 min final extension at 72°C. PCR amplicons were not observed in negative controls (H_2_O) throughout the experiment. The presence of PCR products was determined by analyzing 5 μl of product on 1.0% agarose gel. The triplicate PCR products from each sample were pooled together and purified using Agencourt AMPure XP beads (Beckman Coulter) before being sequenced at the Institute of Biotechnology (University of Helsinki, Finland) using the 454 GS-FLX Titanium protocol (454 Life Sciences/Roche Diagnostics, CT, USA).

#### Sequence data processing

The sequence data were analyzed using the mothur pipeline (v. 1.31.2) [34] following a modified standard operation procedure [35]. Briefly, the raw reads were subjected to quality control and each sequence was screened for a match to the sequencing primer (ITS4) and a valid DNA tag. Sequences were removed if containing: i) ambiguous (N) bases; ii) homopolymers longer than eight nucleotides; iii) average Phred quality score lower than 25. Each sequence that passed quality filtering was truncated to a 200-bp length after primer and tag removal. To remove sequences that were likely due to pyrosequencing errors, the remaining sequences were pre-clustered within a distance of 1 bp using a pseudo-single linkage algorithm implemented in mothur. Potentially chimeric sequences were identified using mothur-embedded UCHIME [36] and removed. Unique sequences were pairwise aligned with Needleman method [37] and the aligned distance matrixes were clustered into operational taxonomic units (OTUs) using the average neighbor algorithm at 97% similarity. All global singletons (OTUs containing only one sequence across all samples) were omitted due to their uncertain origin [38].

To determine taxonomic affinities, a randomly selected representative sequence for each OTU was phylogenetically classified using mothur-formatted copy of the full "UNITE+INSD" datasets version 6 (INSD: International Nucleotide Sequence Database) with an 80% bootstrap confidence threshold in mothur [39]. Finally, the percentage of sequences classified as *Piloderma* sp. (three OTUs, accession numbers LK939126-LK939128) from all detected OTUs were calculated. The sequences of five *Piloderma* sp. from the closest matches in the UNITE+INSD database search [40] were chosen as reference sequences and the ITS2 region of these sequences were extracted using ITS extractor integrated in UNITE. The sequences of the three *Piloderma* sp. OTUs, five *Piloderma* sp. and four other reference strains were aligned and phylogenetic tree was constructed by neighbor-joining bootstrap method in Megan5 [41].

#### Eddy covariance flux measurements and calculation of GPP

The net ecosystem exchange (NEE) was measured using the eddy covariance (EC) technique [42]. Briefly, the measurement system, located 23.3 m above the ground, included an ultrasonic anemometer (Solent Research 1012R2, Gill Instruments Ltd, Lymington, Hampshire, England) for measuring three wind speed components and temperature and a closed-path infrared gas analyzer (LI-6262, LI-COR Biosciences, Lincoln, NE) measuring CO_2_ and H_2_O concentrations. The 30 min averaged NEE was partitioned into total ecosystem respiration (TER) and gross primary productivity (GPP) as described in Kolari *et al*. [43] using half-hourly averaged incident photosynthetically active radiation (PAR) measured above the canopy with quantum sensor (Li-Cor LI-190 SZ, Lincoln, NE). TER was calculated from night-time NEE measurements with an Arrhenius type function [44] using the soil organic layer temperature as the driving force. The temperature dependence of the night time TER was applied to daytime and the 30 min daytime GPP (defined to be always positive) was calculated by subtracting the measured NEE (negative for the net uptake) from the estimated TER (always positive). In case of missing or rejected NEE, the 30 min GPP was calculated from a saturating light response parameterized with the accepted NEE data. The EC tower located in the same forest stand, within 50 m from the three soil sampling areas that were used for microbiological analyses.

#### Chamber measurements

Soil CO_2_ efflux was measured using an automated chamber consisting of a 6-mm-thick PMMA (acryl) box (20 cm × 20 cm × 25 cm in size) covered with aluminum foil. The chamber was placed on an aluminum frame (7 cm) inserted in the litter layer of the soil [45]. The air inside the chamber was mixed continuously during the growth period, and also between measurements, using a small fan with a 2.5 cm diameter. The CO_2_ concentration inside the chamber was recorded with GMP343 diffusion type CO_2_ probes (Vaisala Oyj, Vantaa, Finland). The relative humidity inside the chamber was recorded continuously with a semiconductor sensor (HIH-4000, Honeywell International, Inc.) and the temperature was measured with a thermocouple type K sensor. The top of the chamber was automatically tilted, between measurements, vertically on the side of the frame so that the soil and the vegetation under the chamber remain intact. For the measurements, the box was gently turned back on the frame. All the chambers were closed for 3.5 minutes in every 30 minutes. The data from the automated chambers were recorded with AD converters (Nokeval, Nokeval Oy, Nokia, Finland) at 5 second intervals. The spatial representativeness of the automated chamber was also verified with manual chamber measurements [46] that were conducted biweekly on 14 permanent measurement plots located in the area. The chamber measurement site located in the same forest stand, within 50 m from the three soil sampling areas that were used for microbiological analyses.

#### Soil temperature and moisture

Soil temperatures were recorded at 15-min intervals using silicon temperature sensors (Philips KTY81–110, Philips semiconductors, Eindhoven, the Netherlands) installed in the middle of the organic layer at 3 cm and eluvial horizons at 7 cm depths. The average temperatures within 1 h were used in data analysis. Volumetric water content in respective soil layers was monitored at hourly intervals using the TDR-method with unbalanced steel probes connected to a cable radar (TDR-100, Campbell Scientific Ltd, Logan, Utah). The soil temperature and measurement site located within the same forest stand, not further than 50 m from the three soil sampling areas that were used for microbiological analyses.

#### Statistical analysis

A one-way ANOVA was performed in order to analyze treatment or seasonal effect on protease production, *Piloderma* OTU sequence abundance, biomass, root tip numbers, root to shoot ratio and needle N uptake. The statistically significant differences between the treatments were determined using the Tukey’s post hoc test. If the requirements of ANOVA were not fulfilled, even after transformation of the data (square root, ln or lg10, or arcsin1/10√ for percentage data), the non-parametric Kruskall-Wallis test using pairwise multiple comparison was performed. A P-value <0.05 was set as the limit for statistical significance.

We investigated the possible factors explaining seasonally the abundance of mycorrhizal root tips (in total and *Piloderma* sp.), *Piloderma* sp. OTUs and protease production in mycorrhizal root tips (in all mycorrhizal and in *Piloderma* sp. tips) and soil at the field measurement site in Hyytiälä by studying their correlation with the last seven days gross primary production (GPP) (indicative of recent C assimilation), net ecosystem exchange (NEE), soil CO_2_ efflux, soil water content (SWC) and temperature with Spearman’s non-parametric correlation.

## Results

### Laboratory experiments

#### The effect of N source on fungal growth, nitrogen uptake and biomass production of host plant in symbiosis with *P*. *olivaceum*

*P*. *olivaceum* grew equally well on different N sources in liquid media and demonstrated even a tendency (*P* = 0.113) for a preferential utilization of organic N sources, amino acids and proteins. The biomass in 100% NH_4_Cl-containing media was on average 40.5 mg (standard deviation 31.4), in 90% amino acid-containing media 83.3 mg (28.0) and 90% BSA-protein containing media 111.7 mg (49.8). The biomass production was therefore almost three times higher in cultures where 90% of inorganic N in HA-media was replaced with BSA protein.

**Fig 1:**
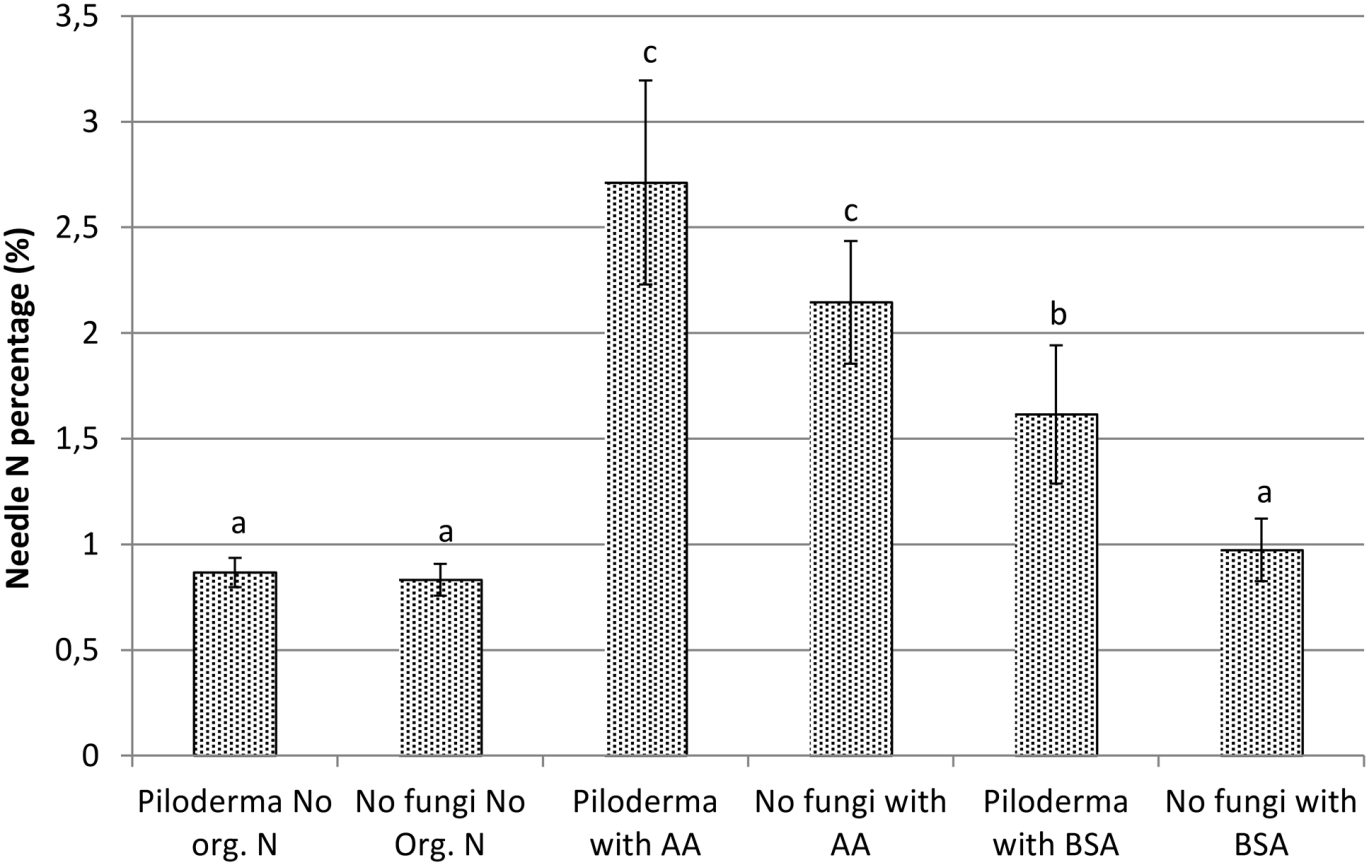
The effect of N source on Scots pine needle N percentage in the presence or absence of *P*. *olivaceum*. The different letters above the standard deviation bars indicate statistical difference (*P* < 0.05).

Sterile Scots pine seedlings and seedlings with *P*. *olivaceum* as an ECM symbiont showed similar abilities to use inorganic N or N from amino acids (Fig 1). However, only Scots pine seedlings colonized by *P*. *olivaceum* were able to utilize BSA as a N source (Fig 1). No significant difference in seedling biomass (g DW shoot, root or root shoot ratio) was observed between mycorrhizal and non-mycorrhizal treatments at any of the fertilization levels. However, root biomass and root/shoot ratio in seedlings colonized by *Piloderma olivaceum* tended to be higher if organic N was available (S3 Table). Note! The seedlings were not under N treatment for their entire growth period (see Materials and Methods).

#### Protease production of pure culture strains

**Fig 2:**
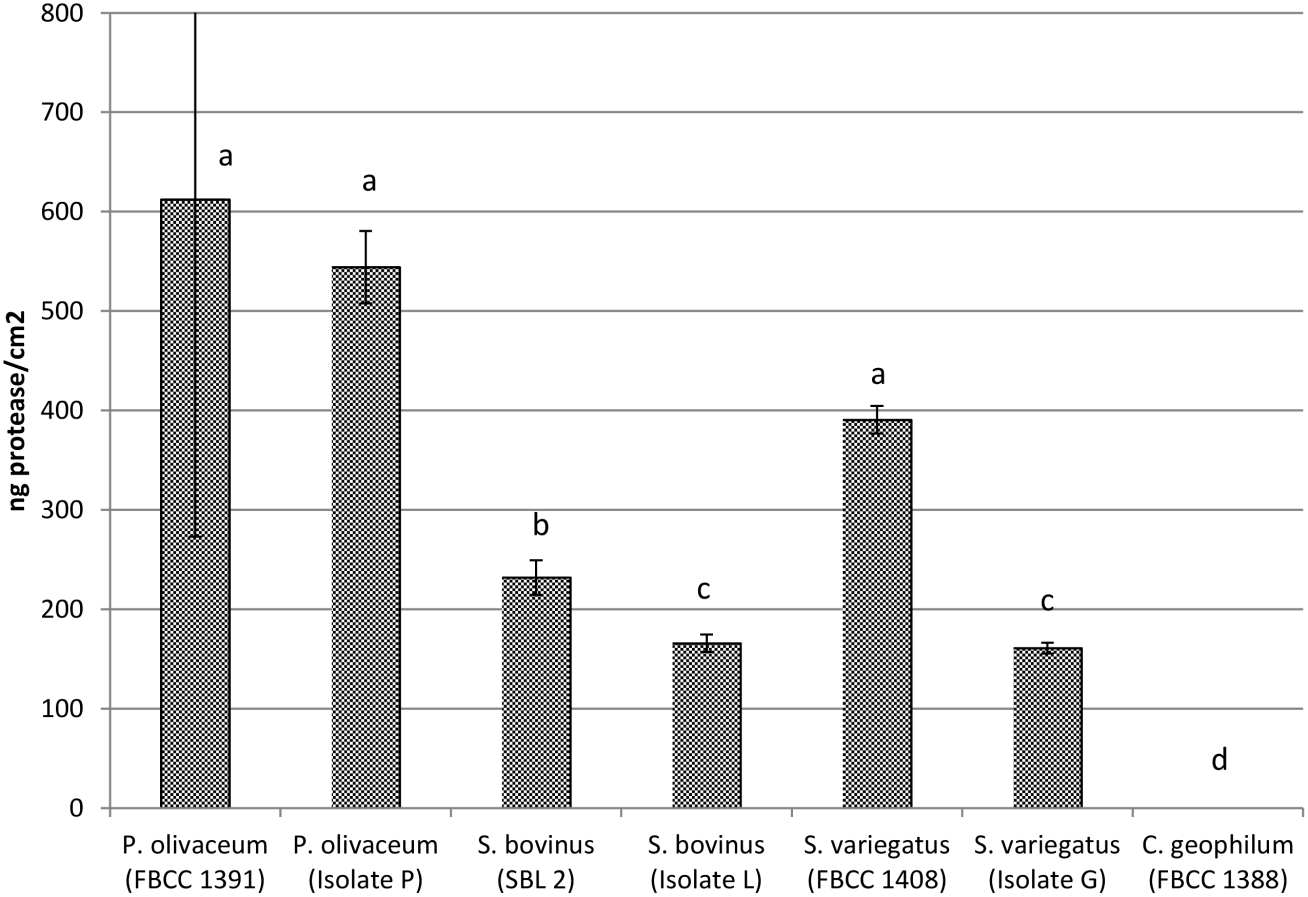
Protease production of ectomycorrhizal culture collection isolates and field isolates from the studied Scots pine roots. For details of the fungal cultures used in this study, see Table 1. Independent-samples non-parametric Kruskal-Wallis test with pairwise comparisons were performed (N = 3). The different letters above standard deviation bars indicate statistical difference (P<0.05), adjusted for multiple comparisons.

*P*. *olivaceum* produced high quantities of protease as measured using the PF0100 Protease Fluorescent Detection Kit (Fig 2). *Suillus variegatus* produced the second highest quantities of proteases, followed by other *Suillus* sp. One of the two *S*. *variegatus* strains produced statistically as much proteases as *P*. *olivaceum*. However, *Cenococcum geophilum* did not show any ability to produce proteases (Fig 2).

### Field experiment

#### Protease production in soil and in ectomycorrhiza per m^2^

**Fig 3:**
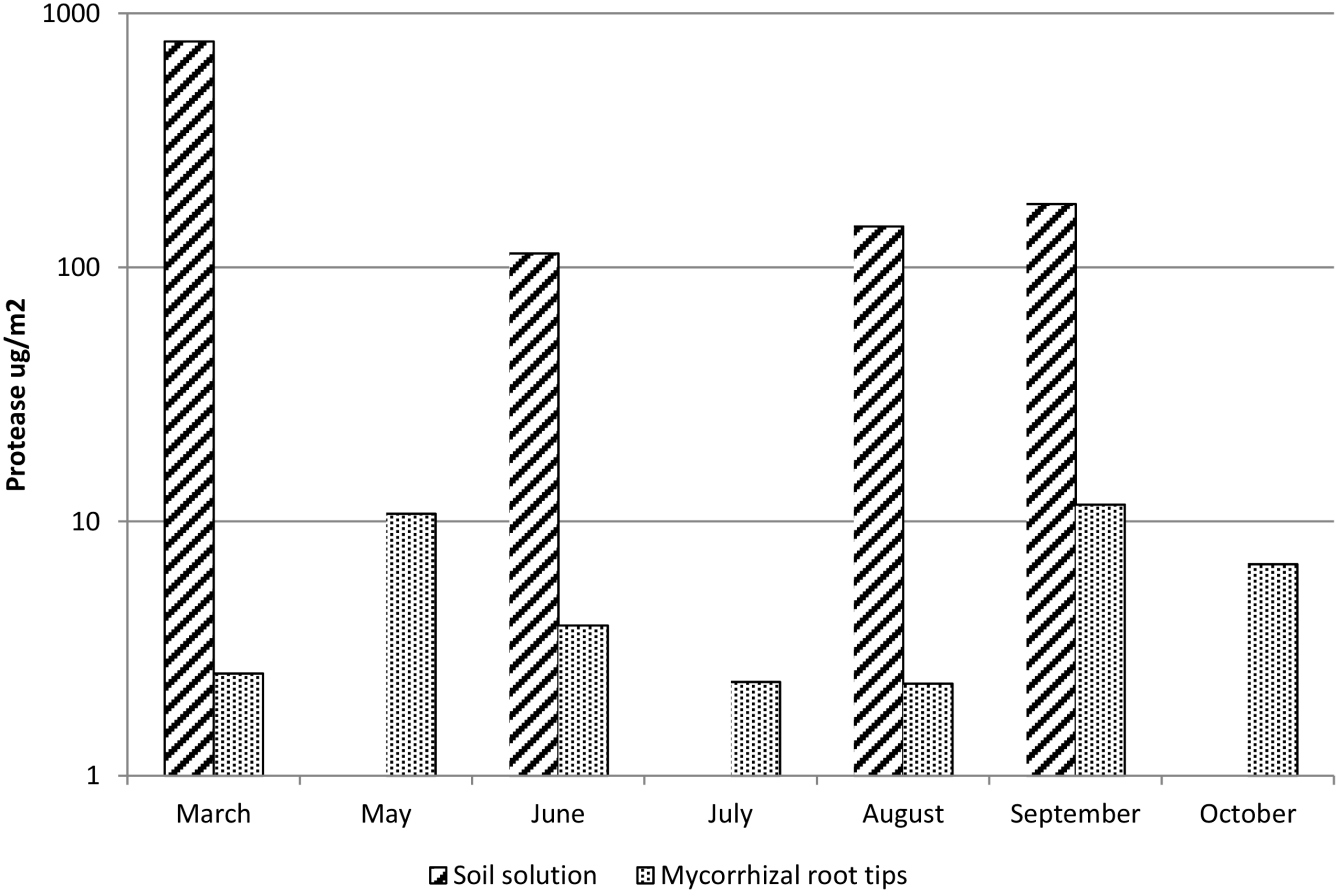
The average protease quantities in soil solution and in all ECM root tips over one growth season. The data was extrapolated to represent protease quantities (μg) per surface of soil (m^2^). No data was available for April. The Y-axis is in a logarithmic scale. Differences between protease quantities in soil solution and ECM root tips or between months were not significantly different due to large variation.

**Fig 4:**
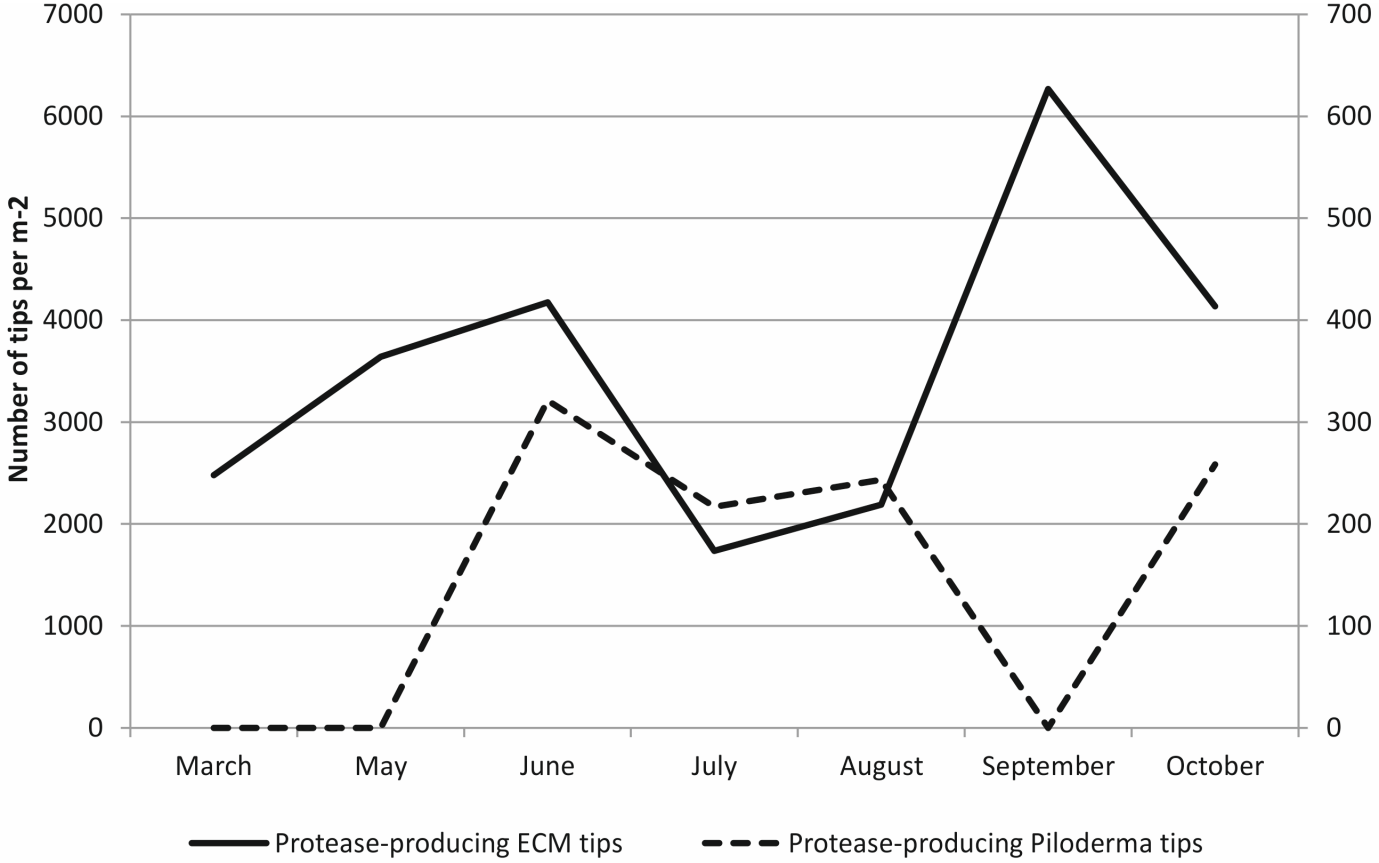
The number of protease-producing ECM root tips (in total) and *Piloderma* sp. root tips from March to October. Data present monthly pooled values from all three replicate sites, no statistical testing performed.

In soil solution, higher protease production per m^2^ forest soil was observed than that in all ECM root tips together: protease production averages in soil were 15–300 times higher than that in ECM tips (Fig 3) but the differences were not statistically significant due to large variation (independent-samples non-parametric Kruskal-Wallis test, *P* = 0.309). However, the production in ECM appeared more stable throughout the year. Protease production in soil was highest in March, under snow cover, whereas the peak of protease production in ECM root tips was in spring and autumn (Fig 3). Protease quantities did not differ significantly between months (independent-samples non-parametric Kruskal-Wallis test, *P* = 0.115 for soil solution, *P* = 0.370 for ECM). The number of protease-producing ECM root tips seemed to peak in early summer and in autumn whereas the number of protease-producing *Piloderma* sp. tips during the summer months (Fig 4). On average, 13% of all ECM root tips showed protease production and this proportion was relatively stable over the growing season.

#### Abundance of *Piloderma* sp. in ectomycorrhiza and in soil

The total number of Scots pine root tips ranged from 20 000 to 41 000 tips m^-2^ (S1 Fig). The yellow *Piloderma* sp. morphotype was the most common morphotype in the roots of Scots pine in March (under snow) but in May it had the lowest abundance (S1 Fig). Approximately 10% of all mycorrhizal root tips belonged to *Piloderma* sp. morphotype between July and October (S1 Fig). The total number of root tip or *Piloderma* root tip were not statistically significantly different between the sampling months (independent-samples non-parametric Kruskal-Wallis test, *P* = 0.864 and *P* = 0.185, respectively).

**Fig 5:**
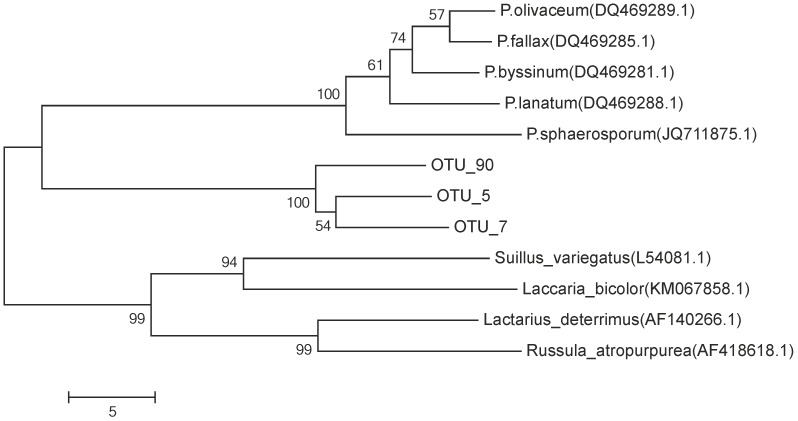
The similarity of ITS2 sequences from the three *Piloderma* sp. OTUs, five *Piloderma* sp. reference and four other reference strains. Accession numbers of reference strains are marked in brackets after species name. Sequences were aligned and phylogenetic tree was constructed by neighbor-joining bootstrap method in Megan5.

The proportion of sequences with the closest similarity to *Piloderma* sp. was determined by amplifying and sequencing the ITS2 region from the humus samples (N = 3), taken from the same soil cores where the morphology and protease production of root tip were recorded. 126 564 sequences were obtained after trimming and quality control (78% of the total 161516 raw sequences), and they were classified into 1403 OTU (2073 OTU with singletons) with 97% similarity. Three OTUs were identified as *Piloderma* sp. (LK939126-LK939128). Using the phylogenetic tree constructed by neighbor-joining on ITS2 sequence, these three OTUs clearly clustered to genus *Piloderma* but the similarity to known *Piloderma* species varied and did not allow accurate identification at species level (Fig 5). The OTUs were therefore classified as *Piloderma* sp. Based on visual observation of forest soil, yellow *Piloderma* sp. is highly abundant in hyphal form in the sampling site.

**Fig 6:**
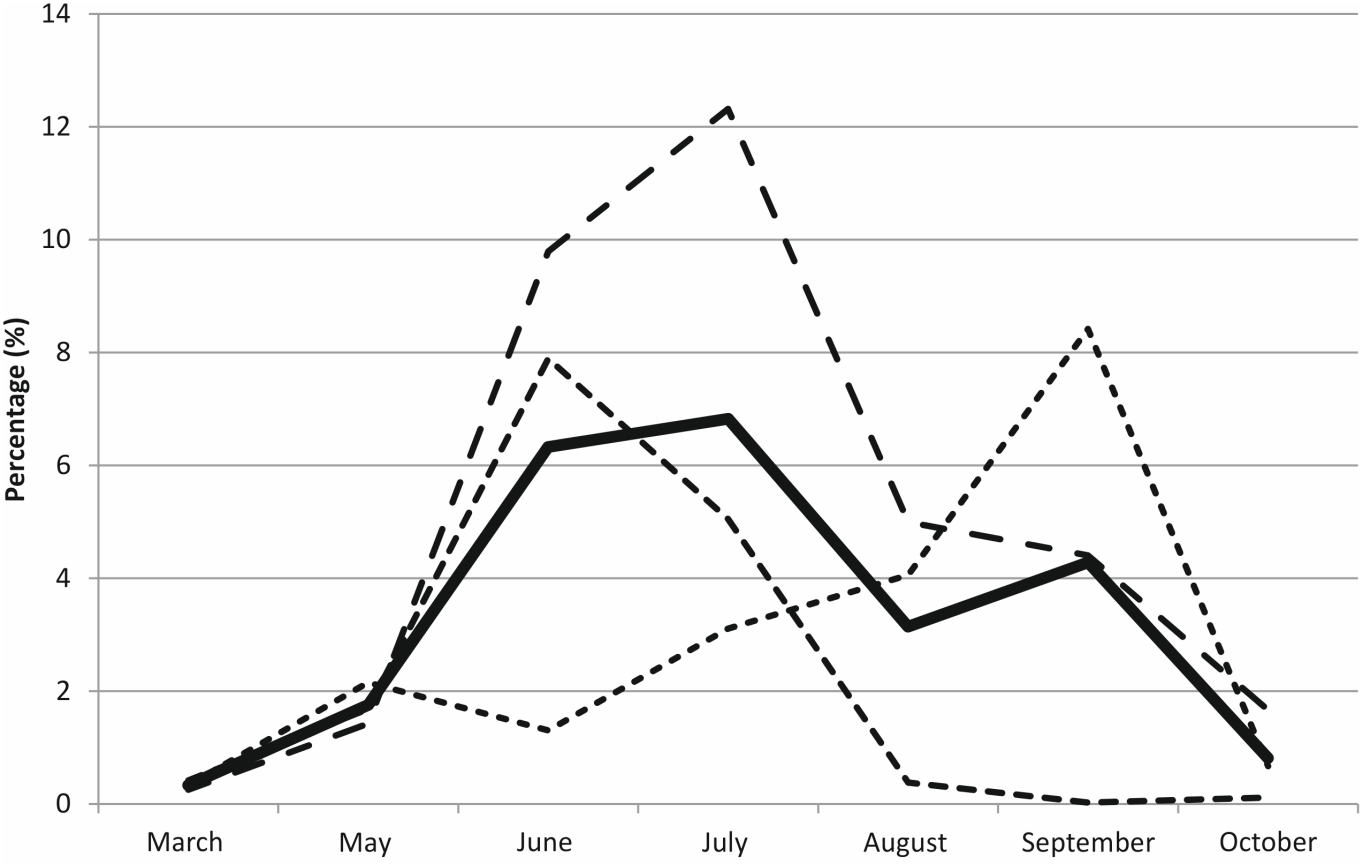
The proportion of *Piloderma* sp. of all the fungal ITS2-region sequences in humus layer. Sequencing was done using 454-pyrosequencing technology. No data was available for April. Differences between sampling months were not statistically significant.

On average, 3.5% of all fungal sequences (range 0 to 12%) belonged to genus *Piloderma* that was the third most common genus in the humus layer in the site (S2 Fig). The seasonal pattern of *Piloderma* sp. OTUs in the soil was similar to that of the protease active *Piloderma* sp. mycorrhiza (Fig 4): the abundance was increasing until July and decreasing from July to October (Fig 6) but the differences were not statistically significant (independent-samples non-parametric Kruskal-Wallis test, *P* = 0.147). *Piloderma* sp. in the soil showed similar seasonal variation with a peak in June and July in two of the three plots, whereas the third plot had the highest proportion of *Piloderma* sp. in September (Fig 6).

#### Gross primary production (GPP) and net ecosystem exchange (NEE)

**Fig 7:**
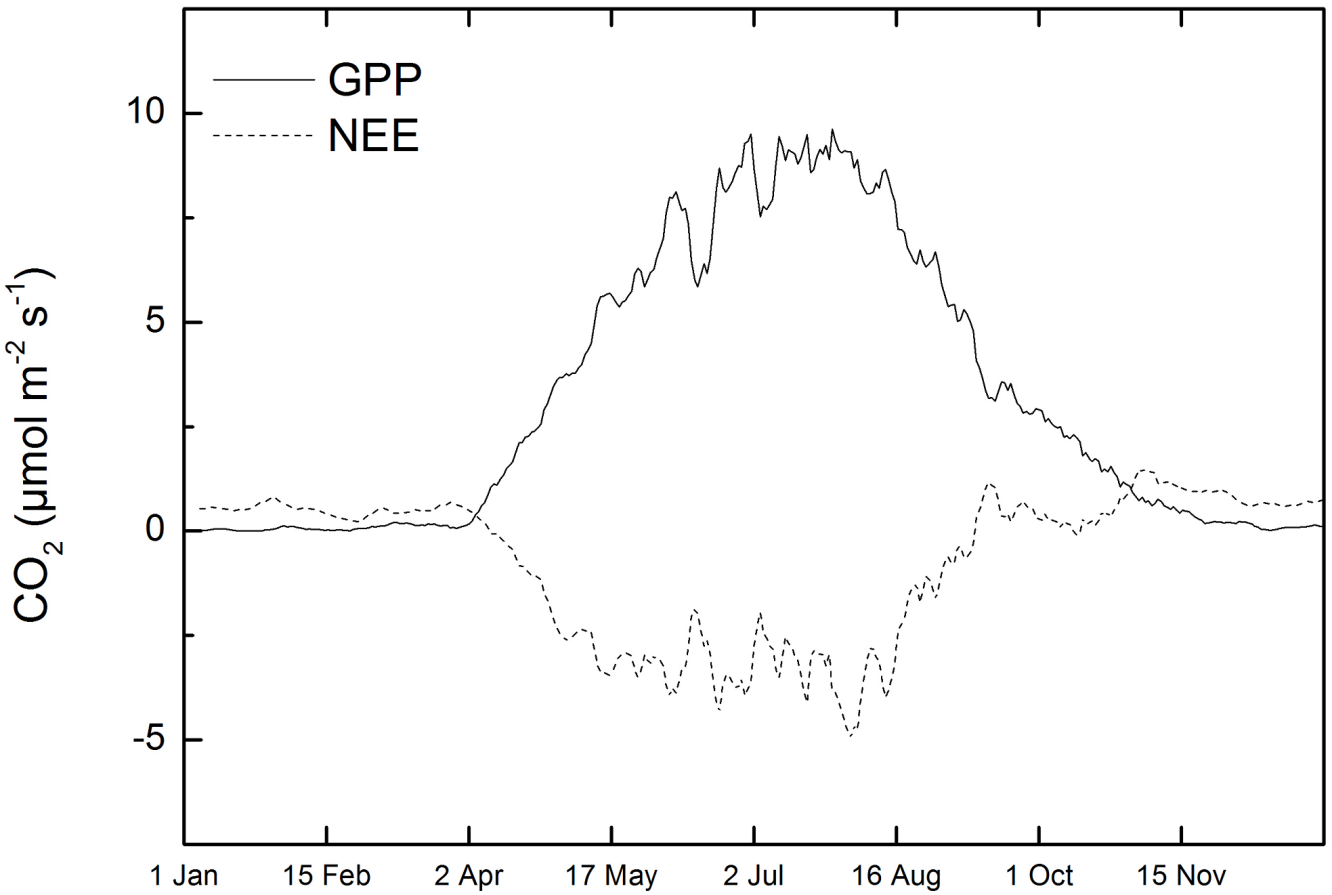
The seasonal pattern of GPP and NEE over the study period at the measurement site in Southern Finland. Positive values in gross primary production (GPP) and negative values in net ecosystem exchange (NEE) indicate CO_2_ assimilation measured with the eddy covariance system over the forest canopy.

Photosynthesis resumed after winter already in late January. The GPP started to increase in late March and continued to reach a maximum of the year (30.4 μmol m^-2^ s^-1^) on June 21^st^ (Fig 7). It remained at high level until the middle of August and started to decline towards the autumn. A similar pattern was observed in NEE (Fig 7).

#### Soil CO_2_ efflux and soil temperature

Soil CO_2_ efflux started to increase in late April and continued increasing until the end of August (12.6 μmol m^-2^ s^-1^, S3 Fig). CO_2_ efflux started to decline on 26^th^ August along with soil temperature towards the autumn except for a peak on 20^th^ September after a long dry period lasting from July to the middle of September (S3 and S4 Figs). The temperature of the soil at the sampling site followed the same pattern as GPP and NEE (S4 Fig, Fig 7).

#### The correlation of site productivity, temperature and moisture with mycorrhizal abundance and protease production

There was significant positive correlation between the last 7-day GPP and the proportion of *Piloderma* sp. of all the OTUs in soil (r^2^ = 0.829, *P =* 0.042) (Table 1). The total number of *Piloderma* root tips and protease-producing *Piloderma* root tips had also a positive but non-significant correlation with the last 7-day GPP (Table 1). There was non-significant negative correlation between total number of ECM root tips, protease-producing root tips and protease in soil solution and GPP. However, total number of ECM root tips and protease-producing root tips had a statistically significant positive correlation with the soil water content of the last 7-days (r^2^ = 0.829, *P =* 0.042 and r^2^ = 0.943, *P =* 0.005, respectively). The *Piloderma* sp. OTUs in soil correlated also positively with the last 7-day soil temperature (r^2^ = 0.829, *P =* 0.042) (Table 1).

**Table 1:** Spearman’s rho correlation of ectomycorrhizal (ECM) root tip numbers (total and genus *Piloderma*), *Piloderma* sequence (OTU) abundance, protease producing ECM root tips (total and genus *Piloderma*) and soil solution protease quantities with forest stand parameters gross primary production (GPP), net ecosystem exchange (NEE), soil CO_2_ efflux, soil water content (SWC) and soil temperature (Temp). Significance levels are in brackets, significant correlations (P<0.05) are marked in bold letters, trend (0.05< P <0.1) in italics.

	Total no of ECM tips	Total no of *Piloderma* tips	Abundance of *Piloderma* OTUs	Protease- producing ECM tips	Protease-producing *Piloderma* tips	Protease in soil solution
GPP^1^	-0.429 (0.397)	0.257 (0.623)	**0.829 (0.042)**	-0.543 (0.266)	0.174 (0.742)	-0.091 (0.864)
NEE^1^	0.314 (0.544)	-0.086 (0.872)	*-0*.*771 (0*.*072)*	0.486 (0.329)	-0.029 (0.957)	0.273 (0.600)
Soil CO_2_ efflux	-0.429 (0.397)	0.543 (0.266)	0.657 (0.156)	-0.486 (0.329)	0.116 (0.827)	0.395 (0.439)
SWC	**0.829 (0.042)**	0.029 (0.957)	-0.371 (0.468)	**0.943 (0.005)**	0.000 (1.000)	0.395 (0.439)
Temp	-0.486 (0.329)	0.600 (0.208)	**0.829 (0.042)**	-0.543 (0.266)	0.058 (0.913)	0.213 (0.686)

^1^ Positive values in GPP and negative values in NEE indicate CO_2_ assimilation.

## Discussion

Our data provides new evidence demonstrating the importance of ECM fungal genera in N uptake by Scots pine in boreal forests using genus *Piloderma* sp. as an example. Our results showed the ability of *Piloderma* sp. to obtain N from proteinaceous sources and provide N to the host plant. *Piloderma* sp. was shown to grow on organic N, including proteins. *Piloderma* sp. strains in culture showed the ability to produce large amounts of proteases, enzymes necessary in breaking proteins into amino acids. Scots pine seedlings were shown in this study to utilize N from BSA protein if in symbiosis with *P*. *olivaceum* confirming the importance of fungal protease production to plant nutrition. In addition to the laboratory evidence, protease production was measured in the field from soil solution as well as on individual ECM root tips, including *Piloderma* sp. morphotypes. Protease production of mycorrhizal root tips in general correlated positively and significantly with soil water content but negatively with temperature and gross primary production (GPP). Interestingly, opposite to the general trend, the quantity of protease-producing *Piloderma* sp. ectomycorrhiza peaked in the summer and correlated positively although non-significantly with GPP of the forest stand, soil respiration and temperature. The proportion of sequences from humus samples, classified as *Piloderma* sp. OTUs correlated positively and significantly with GPP and soil temperature indicating high seasonal pattern of *Piloderma* sp. in soil.

*Piloderma* sp. was found to be very common as symbiotic ectomycorrhizal fungi in the Scots pine boreal forest in the Hyytiälä SMEAR II Forest station, Finland. During the sampling period, on average, 10% of all mycorrhizas were *Piloderma* sp. indicating that this ECM had high overall significance. 3.5% of all fungal hyphal sequences from soil on average belonged to OTUs identified as genus *Piloderma* sp., ranging seasonally from 0.02 to 12%. Given that OTU diversity has been shown to be several thousands in a recent fungal community analysis of boreal forest soil [13], *Piloderma* sp. clearly belongs to one of the most dominant genera at the site (S2 Fig). The average abundance of *Piloderma* sp., both as ectomycorrhiza in root tips during summer months and in hyphal form in soil, correlated positively with the GPP of the host trees, temperature and soil respiration, respectively, but surprisingly not with soil moisture. Our result show evidence that *Piloderma* sp. abundance could be regulated by plant C supply, despite its potential for SOM degradation by oxidative enzyme production [19]. Low abundance of *Piloderma* OTUs in soil in spring and autumn, during moist periods with low GPP, indicate that *Piloderma* sp. may not be able to compete with other soil fungi as a decomposer.

As the abundance of *Piloderma* sp. OTUs in soil and protease-producing *Piloderma* root tips seems to correlate with site productivity, one can assume that organic N uptake from SOM by *Piloderma* sp. occurs during the period of active photosynthesis. Based on the low OTU frequency, it seems likely that the hyphae of *Piloderma* sp. die off during late autumn and winter time, although in general the protease quantities in the soil continue to remain at high levels. This may indicate that other microbes are responsible for soil proteolytic activities when the GPP is low. ECM fungi secrete in majority aspartic proteases whose optimum pH is usually acidic [25, 47] whereas the standard measurement conditions used in this assay were neutral (pH 7.5). Therefore, our results may be underestimates for real protease production, in particular for ECM fungi.

Considering that N-containing chitin is one of the key constituents of fungal biomass, it seems evident that more N is retained in *Piloderma* sp. hyphae when its abundance in soil is high. Accordingly, the biomass-bound N will be available for other soil micro-organisms when host plant C supply is low and the hyphae die. Therefore, *Piloderma* sp. hyphae could form a transient N pool in soil that transforms organic N from SOM to fungal N, consisting of e.g. proteins and chitin. High microbial protease quantities detected in soil solution indicates active utilization of proteins (Fig 3), while chitin is regarded to be more recalcitrant and without chitinolytic microbes practically unavailable to Scots pine as N source. Many ERM fungi that form symbiosis with boreal forest ground vegetation plants are able to utilize and degrade chitin [48]. Some ERM fungi have been shown to be associated also with Scots pine [49–50]. An increase in ECM hyphal biomass in the future high-CO_2_ climate suggests that larger amounts of N will be bound to fungi and maintain strong N limitation in boreal forest ecosystems [51–53]. Similarly to saprotrophic fungi [54], our results suggest across trophy-level re-translocation of N originated from SOM from mycorrhiza to soil decomposing microbes. Rapid turnover of ^13^C-labelled dead ectomycorrhizal fungal biomass and its incorporation into free-living soil fungi was also shown using stable isotope probing [55]. For this reason, the temporal dynamics of soil microbial communities is of crucial importance when estimating plant-microbial costs and benefits and host plant fitness. The fate of hyphal-bound N over longer time scales and the potentially increasing role of ERM or free-living soil fungi with high chitinolytic activities are important questions that will influence soil C and N cycling and accuracy of earth system models [56].

## Conclusion

We show laboratory and field scale evidence that a common ECM fungal genus of Scots pine in boreal forests has the ability to potentially impact soil organic N transformations and host plant N uptake. Our results provide experimental evidence of the ability of *P*. *olivaceum* to capture and provide its host plant with N originating from proteinaceous sources. Our data demonstrate high protease enzyme production by *P*. *olivaceum* in pure cultures. Also at the field scale, *Piloderma* sp. mycorrhiza produces proteases and its abundance as mycorrhizae and as hyphae in soil is high, correlating positively with forest productivity (GPP). Our results therefore provide new evidence for the role of an ECM fungal genus in liberating N from structural proteins in SOM and transforming it to hyphal-bound N, available for other soil microbes after the death of fungal cells. Further research focusing on other ECM genera is needed to be able to estimate the quantitative importance of the studied mechanisms in actual field conditions with diverse fungal communities. *In situ* transcriptomics from environmental samples will in the future be a useful tool in pinpointing functionally active fungal species in soil.

## Supporting Information

S1 Fig The quantity of all ectomycorrhizal (ECM) and yellow root tips belonging *Piloderma* sp. morphotype per m^2^. (TIF)

S2 Fig The most abundant identified fungal genera in the site. The values are average percentages of the genus from all obtained sequences in humus layer over the whole experimental period.
(TIF)

S3 Fig The seasonal pattern of soil CO_2_ efflux at the measurement site during the study period. (TIF)

S4 Fig Soil temperature and soil water content in organic soil horizon at the measurement site during the study period. (TIF)

S1 Table Fungal strains used in the experiment and their accession numbers. (DOCX)

S2 Table The fungal-specific primer pair used in the study. (XLSX)

S3 Table Scots pine plant biomass with or without *Piloderma olivaceum* and with different organic N sources. (XLSX)
