# Supplementary figures and images for: Evidences on the Ability of Mycorrhizal Genus Piloderma to Use Organic Nitrogen and Deliver It to Scots Pine
Source: PLoS One. 2015 Jul 1;10(7):e0131561. doi: 10.1371/journal.pone.0131561 (PMC4489387; doi:10.1371/journal.pone.0131561)

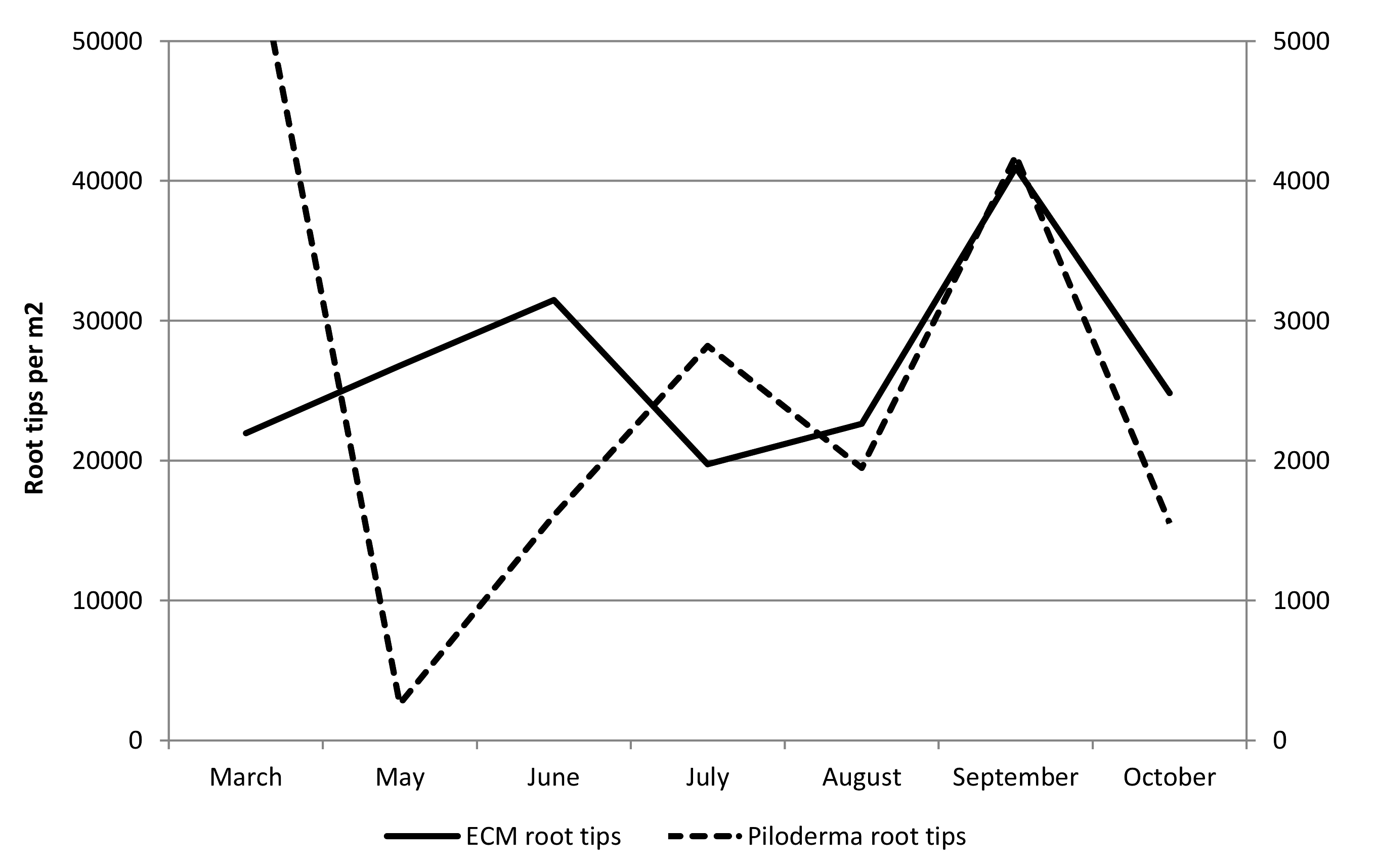

Supplement: S1 Fig — (TIF) [file pone.0131561.s001.tif]

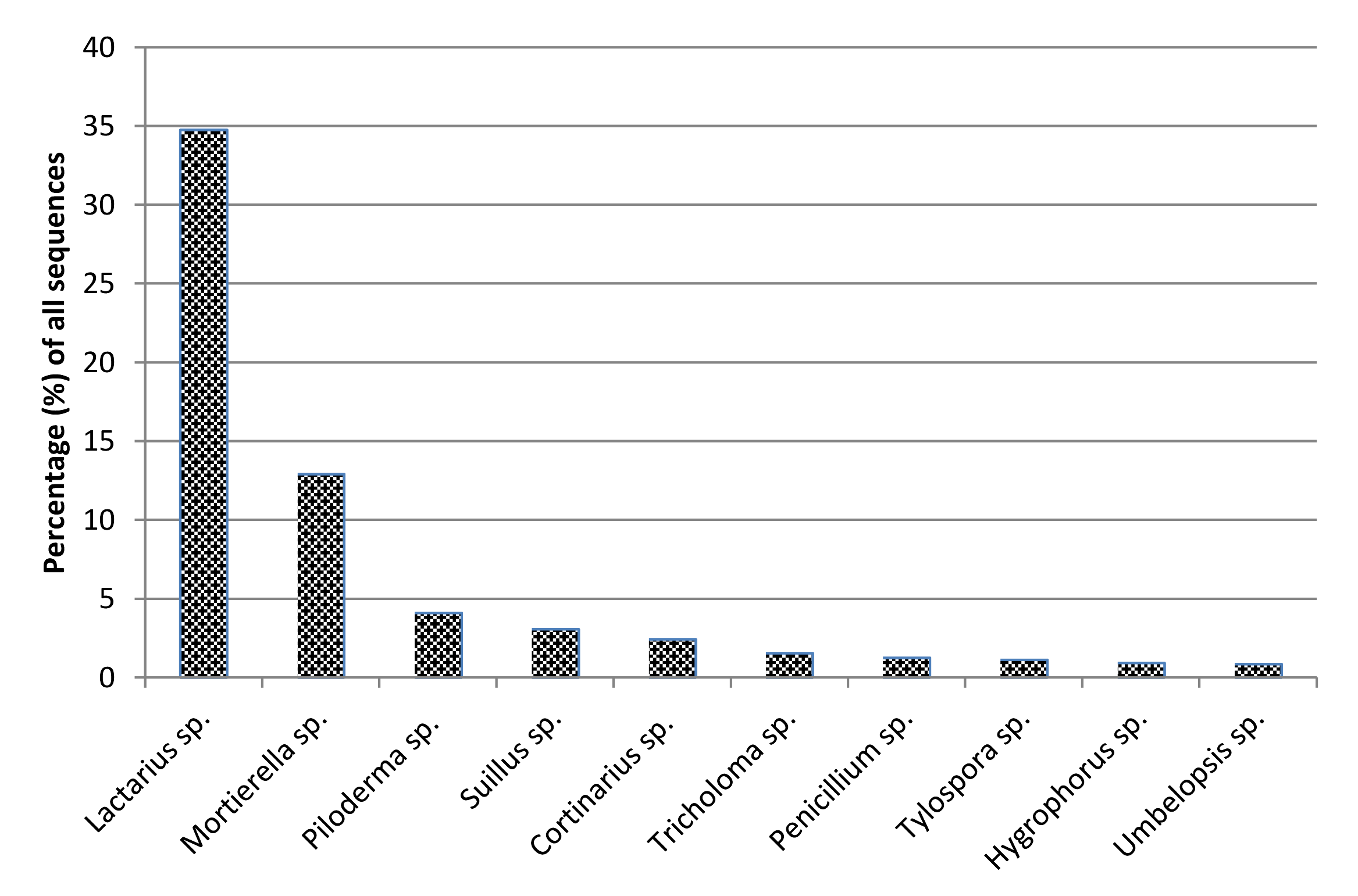

Supplement: S2 Fig — The values are average percentages of the genus from all obtained sequences in humus layer over the whole experimental period. (TIF) [file pone.0131561.s002.tif]

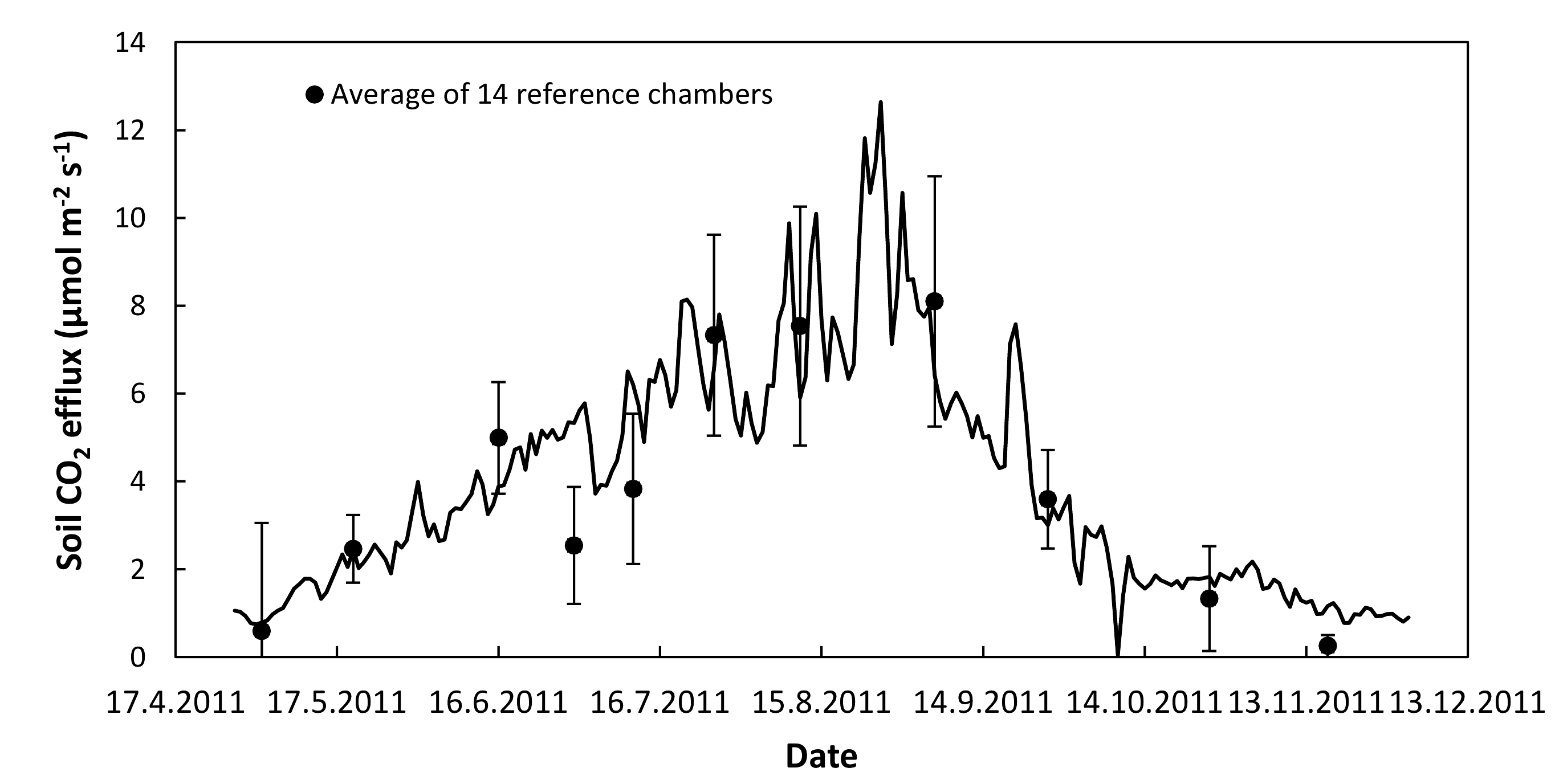

Supplement: S3 Fig — (TIF) [file pone.0131561.s003.tif]

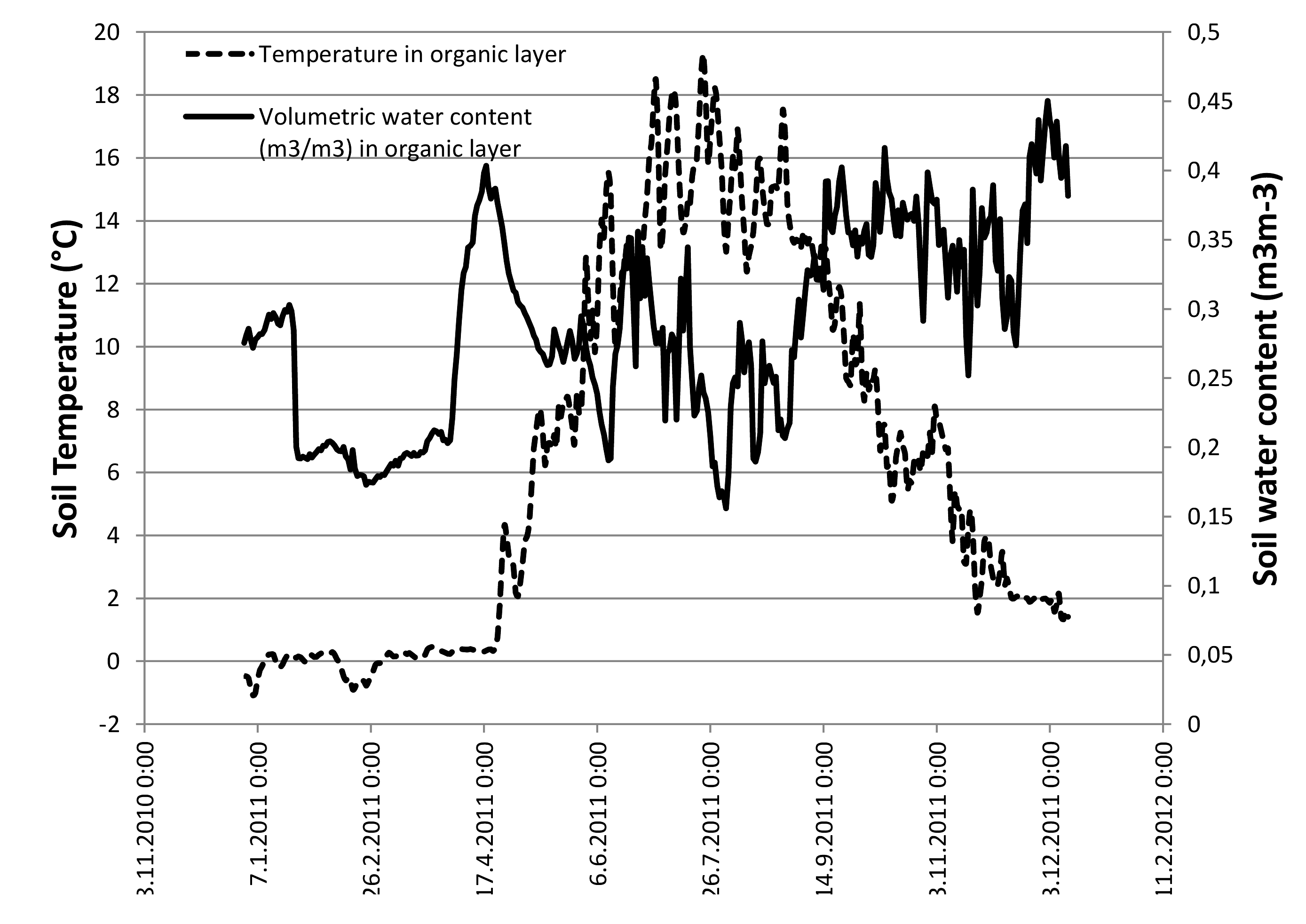

Supplement: S4 Fig — (TIF) [file pone.0131561.s004.tif]
