# Supplementary material for: Evidences on the Ability of Mycorrhizal Genus Piloderma to Use Organic Nitrogen and Deliver It to Scots Pine
Source: PLoS One. 2015 Jul 1;10(7):e0131561. doi: 10.1371/journal.pone.0131561 (PMC4489387; doi:10.1371/journal.pone.0131561)
Supplement: S1 Table — (DOCX) [file pone.0131561.s005.docx]

S1 Table. Fungal strains used in the experiment and their accession numbers and sequence submission of OTUs identified as *Piloderma* sp. (from NGS sequencing). FBCC= Fungal Biotechnology Culture Collection (University of Helsinki, Finland).

| Fungal species | Strain code | Mycelial type^1^ | Stage (early, multi or late) | ITS Sequence  Accession no |
| --- | --- | --- | --- | --- |
| *Piloderma olivaceum (croceum)* | FBCC 1391 | Medium-disctance | late^2^ | AM910819 |
| *Piloderma olivaceum (croceum)* | Isolate P | Medium-disctance | late^2^ | LK052852 |
| *Suillus bovinus* | SBL 2 | Long-distance | early^4^ |  |
| *Suillus bovinus* | Isolate L | Long-distance | early^4^ | LK052848 |
| *Suillus variegatus* | FBCC 1408 | Long-distance | late^3^ | FN556986 |
| *Suillus variegatus* | Isolate G | Long-distance | late^3^ | LK052844 |
| *Cenococcum geophilum* | FBCC 1388 | Medium-disctance | multi^5^ | AM910820 |
| *Piloderma* sp. OTUs in the field |  |  |  | LK939126-LK939128 |
|  |  |  |  |  |

1. Agerer R (2001) Exploration types of ectomycorrhizae: a proposal to classify ectomycorrhizal mycelial systems according to their patterns of differentiation and putative ecological importance. Mycorrhiza 11: 107-114.

2. Herrmann S, Oelmüller R, Buscot F (2004) Manipulation of the onset of ectomycorrhiza formation by indole-3-acetic acid, activated charcoal or relative humidity in the association between oak microcuttings and Piloderma croceum: influence on plant development and photosynthesis. J Plant Physiol 161: 509-517.

3. Finlay RD, Frostegard Å, Sonnerfeldt A-M (1992) Utilization of organic and inorganic nitrogen sources by ectomycorrhizal fungi in pure culture and in symbiosis with Pinus contorta Dougl. ex Loud. New Phytol 120: 105-115.

4. Bowen GD (1994) The ecology of ectomycorrhiza formation and functioning. Plant Soil 159: 61-67.

5. Visser S (1995) Ectomycorrhizal fungal succession in jack pine stands following wildfire. New Phytol 129: 389-401.
